# Perceived Quality of the Mother–Adolescent and Father–Adolescent Attachment Relationship and Adolescents’ Self-Esteem

**DOI:** 10.1007/s10964-019-01007-0

**Published:** 2019-03-19

**Authors:** Renske Keizer, Katrien O. W. Helmerhorst, Loes van Rijn-van Gelderen

**Affiliations:** 1 Erasmus School of Social and Behavioral Sciences, Erasmus University Rotterdam, P.O. Box 1738, 3000 DR Rotterdam, The Netherlands; 2 Research Institute of Child Development and Education - Preventive Youth Care, University of Amsterdam, P.O. Box 15780, 1001 NG Amsterdam, The Netherlands

**Keywords:** Adolescent, Attachment, Fathers, Gender child, Mothers, Self-esteem

## Abstract

There is consensus in the literature that self-esteem stems from relationships with others. In particular, it is assumed that parents play an important role in the development of children’s self-esteem, also in adolescence. Despite the importance of parent–child attachment relationships for adolescents’ self-esteem, we know very little about the extent to which fathers and mothers uniquely contribute to adolescents’ self-esteem. The current study aims to contribute to acquiring knowledge in this research area in three ways. First, by separating the potential influences of father–child and mother–child attachment relationships on sons’ and daughters’ self-esteem, the current study is able to investigate the individual contribution of the father–child and mother–child attachment relationship to female and male adolescent’s self-esteem. Second, by controlling for changes in the quality of the parental relationship and peer relationships the current study is able to isolate linkages between changes in adolescents’ perceived quality of the parent–child attachment relationships and changes in adolescents’ self-esteem. Third, by using longitudinal data and solely analyzing within-person variation, the current study is able to rule out stable confounding factors as alternative explanations. Self-reports of 542 adolescents (mean age at T1 = 13.6 years, percentage female = 0.51) from all three waves of the Dutch cohort study Social Development of Adolescents were used. The longitudinal fixed effects models showed that, for both sons and daughters, changes in the perceived quality of the mother–adolescent attachment relationship and changes in the perceived quality of the relationship between adolescents’ parents were positively linked with changes in self-esteem. Changes in the perceived quality of the attachment relationship with father were only significantly linked to changes in daughters’ self-esteem, not in that of sons. Contrary to the expectations, changes in peer relationships were not associated with changes in adolescents’ self-esteem. These findings suggest that even though adolescents may be increasing their time spent with friends and romantic partners, perceived changes in the attachment relationships with fathers and mothers and in the wider family system are highly important for how adolescents think of and judge themselves.

## Introduction

Self-esteem is an assessment of a person’s feelings of self-acceptance and self-worth (Gecas 1971; Rosenberg 1965). High self-esteem reflects a greater tendency to like, value, and accept oneself (Rosenberg et al. 1995) and has been found to have positive consequences for people’s lives. For example, longitudinal studies have shown that high self-esteem is associated with a diverse range of positive outcomes such as high levels of psychological well-being, physical health, less stressful life events, and more social support (see for overview: Orth et al. 2012). In contrast, low levels of self-esteem have been linked with delinquency, substance abuse, depression, anger, and aggression (e.g., Egan and Perry 1998; Emler 2001). Self-esteem fluctuates during childhood and adolescence; when children reach adolescence levels of self-esteem drop before they gradually rise between middle adolescence to young adulthood (e.g. Orth and Robins 2014; Robins and Trzesniewski 2005).

There is consensus in the literature that self-esteem stems from relationships with others (Sroufe 2002; Thompson 2006). In particular, it is assumed that parents play an important role in the development of children’s self-esteem. Attachment theory indicates that secure attachment relationships promote positive feelings of self-worth and importance (Allen 2016; Sroufe 2002; Thompson 2006). More specifically, the trustworthy warmth of parents provides a foundation for children in infancy to develop mental representations of themselves as loveable and worthy of care. The trust generated by a supportive parent–child relationship provides children with the confidence to explore and engage in new experiences while knowing that the parents’ assistance is available. This theoretical view leads to the assumption that children in secure, supportive parent–child relationships are more likely to perceive themselves positively compared with children in insecure or unsupportive relationships (Thompson 2016). Indeed, data show that children with secure attachment have higher levels of self-esteem during early childhood (for review see: Thompson 2016).

For adolescents, attachment has been defined by a lasting affectionate bond that includes one’s general feelings of security, trust, positive communication, and being supported and accepted in close relationships with others (e.g. Armsden and Greenberg 1987). During adolescence, important developmental changes, such as establishing emotional self-sufficiency, alter attachment relationships with parents. Dependency on parental attachment figures decreases and exploration of new environments takes a central role. With developing cognitive and emotional capacities, adolescents also learn to form attachment relationships with peers and romantic partners (Allen 2016). Nevertheless, it is argued that secure parent–child relationships remain to provide a secure base for adolescents to become increasingly autonomous in order to explore the outside world, which in turn promotes adolescent’s self-esteem (Allen 2016). Empirical evidence shows that attachment relationships with parents indeed serve important functions long after infancy (e.g., Raudino et al. 2013). Specifically, various studies have shown a well-established positive relationship between the quality of parent–adolescent attachment relationships and adolescent self-esteem (e.g., Arbona and Power 2003; Gomez and McLaren 2007; Song et al. 2009; Wilkinson 2004). In sum, adolescents who perceive the attachment relationship with their parents as secure are more likely to have high levels of self-esteem.

Despite the importance of parent–child attachment relationships for adolescents’ self-esteem, it is still an open question whether and to what extent father–child and mother–child attachment relationships uniquely contribute to adolescents’ self-esteem. The current study aims to address this question by investigating the longitudinal relationship between adolescents’ perceived quality of the attachment relationship with their mother and father and adolescent self-esteem. The rationale for looking at the separate influences of father–child and mother–child attachment relationships stems from two theoretical viewpoints. First, attachment theory describes that both infants’ and adolescents’ attachment relationships with their mothers and their father can be different and complementary and thus specific to the parent (Ainsworth 1989; Bretherton 2010; Thomson 2016). Attachment theory indicates that security and exploration represent two sides of the same attachment coin—parents may serve as a haven of safety *and* a trusted companion during exploration. In contrast to this idea, however, several scholars have suggested that mothers and fathers have distinct and complementary attachment roles. Mothers are often viewed as safe haven attachment figures, whereas fathers are considered as facilitators of children’s exploration system (Dumont and Paquette 2013; Grossmann et al. 2002). Previous research has underscored this idea from the perspective of adolescents themselves: Fathers are (seen as) more independence-encouraging, while mothers are more likely to be accepting (e.g., McCormick and Kennedy 1994). Given that most children have spent their entire childhood in a school class with the same children but move to a new school with a new social circle when they reach adolescence (Galambos and Costigan 2003), it is possible that the influence of fathers in their attachment roles of ‘bridges to the outside world’ becomes relatively more important in adolescence. This underscores the need to investigate the individual contributions of father–child and mother–child attachment relationships to adolescents’ self-esteem.

Second, family systems theory assumes that the family as a whole is greater than the sum of its parts and has attributes that cannot be understood simply from the combined characteristics of each family member. The family has a hierarchically organized system, which comprises of smaller subsystems (i.e. the caregiver–child relationship, the couple relationship, the coparenting relationship, and the sibling relationship(s)) that are interdependent and have a continuous and reciprocal influence on one another (Cox and Paley 1997). From this perspective, it is pivotal to incorporate as many subsystems as possible, to be able to obtain an accurate understanding of the influence that the attachment relationship with one specific parent has on adolescents’ self-esteem.

Empirical evidence with regard to links between the quality of maternal and paternal attachment relationships and adolescent’s self-esteem is mixed. The first study on this topic was conducted in the 1980s and found that the attachment relationship with fathers (Gecas and Schwalbe 1986) were more strongly related to self-esteem in adolescents (17–19 years old). In addition, in the study of Paterson et al. (1994), adolescents (aged between 13 and 19 years) reported that their quality of affect toward their mothers and fathers (which is one aspect of attachment) was related to their overall self-esteem. However, utilization of parental support and proximity to fathers (also aspects of attachment) were associated with all three subscales of self-esteem, while this was not true for utilization of parental support and proximity to mothers. In contrast, Noom et al. (1999) found that for Dutch adolescents, ranging from 12 to 18 years old, both fathers and mothers attachment uniquely contributed to self-esteem. Likewise, Arbona and Power (2003) showed that attachment to both father and mother contributed unique variance to self-esteem among high school students of 13–19 years of age and Lui (2008) found that for 12–14 year olds from Eastern Taiwan paternal attachment and maternal attachment had differential influences on adolescents’ social functioning (including self-esteem). Finally, Gomez and McLaren (2007) queried adolescents (age: 18–20 years old) from Australia about their attachment relations with both mother and father and their self-esteem and found in separate analyses for both parents that the quality of the attachment relationship was associated positively with self-esteem. Taken together, most studies show that both maternal and paternal attachment relationship (uniquely) contribute to self-esteem. However, all these studies relied on cross-sectional data and were often unable to control for influences from the wider family system and peers—important limitations as will be addressed further on in this article.

For a comprehensive understanding of linkages between the perceived quality of father–child and mother–child attachment relationships and adolescents’ self-esteem, it is important to assess whether these linkages differ between sons and daughters. Not only are there gender differences in attachment relationships with mothers and fathers—adolescent girls for example report higher quality of attachment to both their mother and father than adolescent boys (Buist et al. 2002; Choi et al. 2012), but Gender Theory (e.g., West and Zimmerman 1987) suggests that gender is central to family roles and behavior of both parents and children. It is argued that boys and girls will be treated differently because boys will be socialized into masculine gender roles while girls will be socialized into feminine gender roles. For example, it is thought that parents will promote more autonomy in boys compared to girls. The gender intensification hypothesis argues that during adolescence, gender differences increase and same-gender parent–adolescent dyads become (even more) different from opposite-gender parent–adolescent dyads (Hill and Lynch 1983). The current study will therefore also investigate whether the relation between the perceived quality of the attachment relationship with fathers/mothers and adolescents’ self-esteem differs by adolescents’ gender.

Differences between opposite-gender parent–adolescent dyads versus same-gender parent–adolescent dyads have already been studied when looking at the cross-sectional relation between adolescent parent–child attachment and adolescents’ self-esteem. One study found evidence for the “same-sex hypothesis”, suggesting that the quality of father–child attachment relationships is more strongly related to self-esteem for boys, whereas the quality of mother–child attachment relationships is more strongly related to those for girls (Song et al. 2009). In contrast, another study showed that father–child attachment relationships predicted self-esteem better than did mother–child attachment relationships for early adolescent girls (Liu 2008). One explanation for these conflicting findings could be that these studies used different questionnaires to measure parent–adolescent attachment (i.e. Inventory of Parent and Peer Attachment of Armsden and Greenberg (1987) versus an adapted version of the Relationship Questionnaire-adolescents of Liu (2008)). An alternative explanation might be that these studies differed in how well they were able to isolate the impact of parent–child attachment relationships on adolescents’ self-esteem. The study by Song et al. (2009) was able to control for some peer influences and was able to take differences by education and age groups into account, whereas the study of Liu (2008), up to our knowledge, did not include any controls.

To accurately assess influences of the perceived quality of father–child and mother–child attachment relationships on adolescents’ self-esteem, it is important to control for influences from the wider family and social context (e.g., Luciano and Orth 2017). The current study controls for the quality of the parental relationship and peer influences. The argumentation to include information on the parental relationship quality is twofold, and is grounded in the aforementioned family system theory. According to this theory, the family has a hierarchically organized system, which comprises of smaller subsystems that are interdependent and have a continuous and reciprocal influence on one another. In this light, it is important to control for the influence of the quality of the parental relationship, to be able to accurately assess the unique contributions of the quality of the father–child and mother–child relationship to adolescents’ self-esteem. First, parents’ relationship quality could influence adolescents’ self-esteem directly; witnessing the conflicts between one’s parents may lead to negative self-views, as children tend to consider themselves as causes of conflicts and blame themselves for a disharmonious marital relationship (Grych et al. 2000). Second, being confronted with numerous fights between one’s parents (i.e. low parental relationship quality) could lower the perception of the quality of the relationship the adolescent has with both of his/her parents.

It is just as important to control for peer influences. During adolescence, children increasingly rely on their peers, especially on close friends, for companionship, intimacy, and support (Brown and Larson 2009; De Goede et al. 2009). One important function of peers in adolescence is thought to be to support the individuation process related to developing independence from parents and developing a separate identity. As such, belonging to a peer group, having a best friend and having a romantic partner might become particularly salient for feelings of self-esteem in adolescence (Rubin et al. 2006). Studies have shown that peers have indeed a strong influence on adolescents’ self-esteem: it has been found that adolescents who have friends (e.g., Bagwell et al. 1998; Bishop and Inderbitzen 1995) and who have friendships of high quality (e.g., Keefe and Berndt 1996; Kingery et al. 2011) show higher self-esteem, both concurrently as well as over time. In addition, studies have shown that beginning a relationship increases self-esteem, whereas experiencing a relationship break-up decreases self-esteem, although this effect was found to disappear after 1 year (Luciano and Orth 2017). Furthermore, having a warm and supportive romantic partner leads to increases in self-esteem over time (Seiffge-Krenke 2006; Murray et al. 2000). In this light, peer relationship may act as protective factors for adolescents’ self-esteem. In addition, having close friends/a best friend may act as a buffer in times when the perceived quality of the father–child (or mother–child) attachment relationship is low. In such times, having a best friend may provide a place of belonging, improve social skills, and/or providing positive feedback and help. This may maintain the adolescent’s feelings about positive value as a person and keep their working models of attachment stable even though their relationship with (a) parent(s) is perceived as to be of low quality.

One of the aims of the current study is to provide a comprehensive understanding of the impact of the perceived quality of the attachment relationship with father and mother on adolescents’ self-esteem. In order to be able to make any causal claims, it is not sufficient to solely control for concurrent changes in the quality of the parental relationship and peer relationships; it is essential to account for as many sources of bias as possible. Each individual has its own stable characteristics, for example temperament, gender, genetic make-up, or ethnicity. It is assumed that these stable individual characteristics may also impact or bias the predictor (the perceived quality of the parent–child attachment relationship) and/or outcome (adolescent’s self-esteem) variables in the study and that it is, therefore, important to control for them. Often, unfortunately, the controls that can be included in analytical models fail to capture *all* of the relevant stable characteristics of adolescents and their family and peers, which leads to biased estimates. One statistical approach that has been used to reduce the threat of omitted variables involves longitudinal fixed effects models (Liker et al. 1985). Longitudinal fixed effects models control for the influence of stable (time-invariant) omitted variables by comparing individuals to themselves overtime, which holds these characteristics constant (Raudenbush and Bryk 2002). They are designed to study the causes of changes within a person. A time-invariant characteristic cannot cause such a change, because it is constant for each person. Not only from a statistical point of view, but also from a substantive one, the choice for the use of longitudinal fixed effects models in the current study makes sense. In a transitional stage in which adolescents undergo various developmental changes, it can be argued that it is highly important to focus on the impact of *changes* in the perceived quality of the parent–child attachment relationship on changes in adolescents’ self-esteem.

## The Current Study

Even though high quality parent–child attachment relationships are important for adolescents’ self-esteem, knowledge is limited when it comes to answering the question whether and to what extent fathers and mothers uniquely contribute to adolescents’ self-esteem. The current study aims to contribute to acquiring knowledge in this research area in three ways. First, by separating the potential influences of father–child and mother–child attachment relationships on sons’ and daughters’ self-esteem the current study is able to investigate the individual contribution of the father–child and mother–child attachment relationship to female and male adolescent’s self-esteem. Second, by controlling for changes in the perceived quality of the parental relationship and peer relationships the current study is able to isolate linkages between adolescents’ perceived quality of the parent–child attachment relationships and changes in adolescents’ self-esteem. Third, by using longitudinal data and solely analyzing within-person variation, the current study is able to rule out stable confounding factors as alternative explanations. Given mixed empirical findings, the current study explores how changes in the perceived quality of the father–child and mother–child attachment relationships influence changes in sons’ versus daughters’ self-esteem, after controlling for (changes in) the perceived quality of the parental relationship, the number of close friends, the presence of a best friend, and the presence of a romantic partner.

## Method

### Sample

Participants were draw from the Social Development of Adolescents (SODA) study (Overbeek et al. 2010). This study was initiated in 2004/2005 (T1) to increase the understanding of the determinants of adolescents’ social development. The second wave of data collection (T2) took place in 2006 and the third wave of data collection (T3) in 2007.

The sample was attained by using a stratified sample procedure. At the start of the study, 28 secondary schools in a 100-kilometre radius around Nijmegen (a medium-sized city in the Netherlands) were contacted and 23 schools (82% uptake rate) decided to participate. The research team, together with the school administrations, decided which and how many classes would be selected in each school. This led to a sample of 2475 adolescents at T1. At T2, 1419 adolescents (57%) still participated and at T3, 774 adolescents (31%) took part in the study. The main reason for dropout was that most of the students who were included at T1 had graduated and left school. In most Dutch secondary schools, class composition—in terms of students following lessons together—changes quite drastically across the years. Thus, within schools the research team was unable to retain all T1 students in the longitudinal sample, as many were transferred to other classes. The high attrition rate, then, can be explained by the school boards’ active replacement of students across classes in a school year rather than students’ active refusal to participate in the study. To acquire an optimal sample size, it was decided to only included classes at T2 and T3 in which at least seven students were present who had also participated in the first wave. This led to a total number of 774 (31 percent) students still present at T3. Logistic regression analyses with dropout as dichotomous dependent variable showed that no selective attrition occurred across the two-year time interval between T1 and T3 with regard to gender, age, educational level, parent–child attachment relationships and self-esteem.

To select the final sample for the current study, the current study used several selection criteria. First, only adolescents were selected whose families were still intact at T1, as questions concerning the relationship quality of the parents were only asked to children who lived with both their parents. Information about family structure was available for of 693 adolescents, including 71 adolescents with divorced parents and six adolescents who had experienced a parental death. Thus, 616 adolescents lived in intact families at T1. Second, it was checked whether adolescents experienced any family transitions during T2 and/or T3. Forty-nine of the 616 adolescents in intact families at T1 encountered a parental divorce or a parental death during the duration of the study. Given the small number, the likelihood that these events had a major impact on the well-being of these adolescents, and the expectation that parent–child attachment relationships may have a substantially different impact on these adolescents’ self-esteem, these students were excluded from the analyses.

Finally, only adolescents with a Dutch origin were assigned to the final sample. At T1, all adolescents were asked to specify in which country their parents were born (possible answer categories: Dutch, Antilles/Aruba, Morocco/Turkey, and Other). It turned out that there was only a small group of non-Dutch adolescents (*n* = 43). Due to the fact that the number of respondents for each subgroup group was quite low, in the current study it wasn’t possible to conduct analyses on the different groups. As the interpretation of any effects of having foreign-born parents would be difficult because of the highly heterogeneous group, it was decided to exclude children with foreign-born parents. This yielded an analytic sample of 542 Dutch adolescents.

The final sample included 276 girls and 266 boys, with a mean age at T1 of 13.59 (*SD=* .03; min-max = 11–16 years). Concerning the educational level, 276 students (51.0 percent) followed lower vocational education programs, and 266 students (49.0 percent) were enrolled in middle or higher-level education programs.

### Procedure

From January to March 2005, questionnaires were administered to the adolescents by undergraduate students involved in the doctoral thesis program. All of these students were given instructions regarding the content of the questionnaire and the administration procedure in a classroom situation. Adolescents filled in the questionnaire in their class during a regular lesson (45–50 min) at school. Both adolescents and parents were informed about the content and purpose of the study. All parents agreed in the participation of their children, but some students called in sick, and thus, missed the questionnaire assessment. In most classes, a teacher was available to assist the undergraduate in distributing the questionnaires among the students and keeping order. It was explained to the adolescents that they were not allowed to talk about their answers in the questionnaires with other students and it was guaranteed that their information would not be shared with a third party (i.e., teachers or parents). After the data collection was finished, research reports were sent to each school on the social development of all participating adolescents, (without providing information that identified individual adolescents). For the T2 (2006) and T3 (2007) data collection, identical procedures were followed.

### Measures

#### Self-esteem

At each wave, adolescents’ self-esteem was assessed using the Dutch version of the Rosenberg Self-esteem scale (RSES; Van der Linden et al. 1983; Rosenberg 1979). This self-report measure includes 10 statements about one’s self-evaluation. Examples items are: *Sometimes I definitely feel useless* or *In general I am satisfied with myself*. Answer categories range from 1 = *Does not fit me at all* to 4 = *Fits me well*. Cronbach’s alpha was 0.86 at T1, 0.83 at T2, and 0.88 at T3. Numerous studies have found support for the construct and concurrent validity of the RSES (e.g., Ghaderi 2006; Westaway et al. 2003). Test–retest reliability has been shown to be adequate (Salyers et al. 2001).

#### Perceived quality of parent–child attachment relationship

The Inventory of Parent and Peer Attachment (IPPA; Armsden and Greenberg 1987) was used at each data collection wave to obtain information on the quality of the attachment relationship between the adolescent and his or her father/mother. The original scale was designed to assess both the affective and cognitive dimensions of attachment security and trust in the accessibility and responsiveness of attachment figures. Please note that only the part on the perceived quality of the parent–child attachment relationship was incorporated in the SODA-questionnaire. The perceived quality of child–peer attachment relationships was unfortunately not included. This issue will be discussed in detail in the Discussion section of this article. In the current study, adolescents completed a shortened version of the Inventory of Parent and Peer Attachment (Armsden and Greenberg 1987). The shortened scale consists of 12 items, such as *When I am angry about something, my father/mother tries to be understanding, If my father/mother knows something is bothering me, he/she asks me about it, and I get easily upset with my father/mother*, and was assessed on a 5-point Likert-type scale format (1 = *almost never or never true*; 5 = *almost always or always true*). Items were recoded to make sure that higher scores reflect a higher quality of the parent-child attachment relationship. For each wave, a mean score was then calculated. For the father-child attachment relationship, Cronbach’s alpha was 0.84 at T1, 0.83 at T2, and 0.88 at T3. For the mother–child attachment relationship, Cronbach’s alpha was 0.81 at T1, 0.82 at T2, and 0.86 at T3. Correlations of 0.66 and 0.64 were found at T1 between attachment to father and attachment to mother for girls and boys respectively.

#### Perceived parental relationship quality

This scale was developed especially for the SODA data collection. Seven items were used: (1) In general, my parents have a good relationship, (2) the relationship between my parents is so bad, that I think it would be better if they separate, (3) my parents do not talk a lot to each other, (4) my parents often fight with each other, (5) when my parents fight, they also resolve their issues, (6) my parents often do fun things together, (7) I often see my parents cuddling. Answers to each question ranged from 1 (*strongly agree*) to 5 (*strongly disagree*). Answers were recoded so that higher scores indicate higher parents’ relationship quality. For each wave, a mean score was then calculated. Cronbach’s alpha for this scale is 0.82 at T1, 0.83 at T2 and 0.82 at T3.

#### Number of close friends

At each wave, the respondents were asked the question: *How many good friends do you have?*

#### Presence of best friend

Also at all waves, the respondents were asked the question: *Do you have a best friend?* Answers were 1 = *yes*, 0 = *no*.

#### Presence of romantic partner

Whether or not a romantic partner was present was measured at each wave by asking the question: *Do you have a boyfriend/girlfriend at the moment?* Answers were 1 = *yes*, 0 = *no*.

### Analytic plan

The current article takes advantage of the SODA’s longitudinal design to estimate repeated-observation fixed effects models of the effects of perceived quality of the attachment relationship with father and mother on adolescents’ self-esteem. In order to construct these models, the data were reshaped into a long, or panel, format, in which the unit of analysis is the respondent/observation. In this reshaped panel file, each adolescent has an observation from the first, second and third survey rounds. The fixed-effects panel models make it possible to isolate linkages between the perceived quality of the attachment relationship with father and mother and adolescents’ self-esteem by focusing on changes in the perceived quality of the attachment relationships between observations. In essence, the models are estimating the way changes in the perceived quality of the attachment relationship with father and mother are related to changes in adolescents’ self-esteem, net of time-invariant characteristics of the family members (e.g. ethnicity, temperament) and time-varying controls (e.g. changes in the quality of the parental relationship and changes in peer relationships).

These fixed-effects panel models take the following form:

$$y_{it} = \beta _1X_{it} + \beta _2X_{it} + \beta _3X_{it} + \alpha _i + u_{it}$$

where *y*_*it*_ is a time-varying measure of adolescents’ self-esteem; *β*_1_*X*_*it*_ is a time-varying variable representing the perceived quality of the attachment relationship with father, *β*_2_*X*_*it*_ b3it is a time-varying variable representing the perceived quality of the attachment relationship with mother; *β*_3_*X*_*it*_ is a vector of this study’s time-varying controls perceived quality of the parental relationship, number of close friends, presence of best friend and presence of romantic partner. α_*i*_ is the unknown intercept for each entity, in other words: the respondent fixed-effect term. This respondent fixed effect is equivalent to a dummy variable for each of the respondents in the analysis; it controls for all observable and unobservable time-invariant characteristics of the respondent, his/her families and peers. Finally, *u*_*it*_ is the error term.

The two main methods of dealing with α_*i*_ are to make the random effects or fixed effects assumption. A fixed effects approach utilizes only the within variations (the over-time changes in the values of variables for an individual) but not the between variations (the differences in the levels of variables across individuals) in estimation. The disadvantage of a fixed effect model compared to a random effect model is that it is not possible to examine the impact of time-invariant characteristics. Random effects models are more precise than fixed effects models, precisely because they enable testing the effects of each included time-invarying control variable. However, if the time-invarying control variables that are included in the random effect models fail to capture *all* of the relevant characteristics of adolescents and their family and peers, random effect models may be biased.

In preliminary analyses Hausman (1978) tests were ran to determine the plausibility of the fixed versus random effects model. The results showed that the estimates would suffer, albeit only mildly, from omitted variables bias and that the use of fixed effects model was preferred. This is why fixed effect models were reported in this article.

All models are estimated in Stata (StataCorp, L. 2009). In preliminary analyses, it was tested whether the assumption of homoskedasticity (constant variance) was met. The user-written programme xttest3 was used to test this. Xttest3 calculates a modified Wald statistic for groupwise heteroscedasticity in the residuals of a fixed effect regression model. The test revealed that the assumption of homoscedasticity was violated. Therefore, the Stata “cluster” subcommand was used to obtain heteroskedasticity-robust standard errors (also known as Huber/White or sandwich estimators). As such, this study’s fixed-effects models uses Stata’s “xtreg, fe robust” command.

The independent variables were entered into the analyses in three steps. The first model only includes the main effects of changes in the perceived quality of the father–adolescent and the mother–adolescent attachment relationship. In the second model only changes in the perceived parental relationship quality, changes in the number of close friends, changes in the presence of a best friend and changes in the presence of a romantic partner were included. The third, and final model, includes all abovementioned variables. To compare nested models and to test whether adding predictors to a model improves the model fit to the data significantly, Likelihood ratio-tests were performed. As preliminary analyses revealed that the effects were significantly different for male compared to female adolescents, the models were run separately for these two groups. To test whether any of the observed differences between male and female adolescents were significant, interaction effects were ran. The results of these interaction effects will also be reported below.

## Results

### Descriptive results

Tables 1 and 2 show the means and standard deviations of the variables in the models across waves for adolescent daughters and sons respectively. In addition, these tables report the outcomes of the one-way repeated measures ANOVA tests that were ran in order to determine if there were significant differences in the main variables in our models over time.

**Table 1:** Descriptive statistics for daughters at T1, T2 and T3 for self-esteem, perceived father-adolescent and mother-adolescent attachment, perceived parental relationship, number of close friends, presence of best friend and romantic partner

Variables	Daughters (*N* = 276)			*F*-test	*p*
	Time 1	Time 2	Time 3		
	*M* (SD)	*M* (SD)	*M* (SD)		
Self-esteem	3.08 (0.56)	3.04 (0.57)	3.07 (0.57)	*F* (2,814) = 2.78	0.065
Perceived quality father-adolescent attachment relationship	4.19 (0.67)	4.09 (0.75)	4.16 (0.77)	*F* (2,814) = 3.43	0.033
Perceived quality mother-adolescent attachment relationship	4.54 (0.64)	4.54 (0.66)	4.64 (0.71)	*F* (2,814) = 6.20	0.002
Perceived quality parental relationship	4.20 (0.56)	4.13 (0.58)	4.09 (0.67)	*F* (2,814) = 5.13	0.006
Number of close friends	13.76 (12.00)	16.33 (19.56)	13.49 (10.23)	*F* (2,814) = 2.98	0.052
Presence of best friend	0.91 (0.29)	0.85 (0.36)	0.83 (0.37)	*F* (2,814) = 4.87	0.008
Presence of romantic partner	0.13 (0.34)	0.16 (0.37)	0.26 (0.44)	*F* (2,814) = 11.14	0.000

**Table 2:** Descriptive statistics for sons at T1, T2 and T3 for self-esteem, perceived father–adolescent and mother–adolescent attachment, perceived parental relationship, number of close friends, presence of best friend and romantic partner

Variables	Sons (*N* = 266)			*F*-test	*p*
	Time 1	Time 2	Time 3		
	*M* (SD)	*M* (SD)	*M* (SD)		
Self-esteem	3.30 (0.47)	3.28 (0.52)	3.33 (0.49)	*F* (2,781) = 2.66	0.071
Perceived quality father–adolescent attachment relationship	4.33 (0.69)	4.22 (0.71)	4.19 (0.74)	*F* (2,781) = 5.10	0.006
Perceived quality mother–adolescent attachment relationship	4.53 (0.60)	4.42 (0.66)	4.50 (0.70)	*F* (2,781) = 3.51	0.031
Perceived quality parental relationship	4.24 (0.50)	4.21 (0.49)	4.15 (0.57)	*F* (2,781) = 4.58	0.011
Number of close friends	16.53 (17.41)	18.37 (19.94)	19.73 (26.67)	*F* (2,781) = 2.27	0.105
Presence of best friend	0.85 (0.36)	0.82 (0.39)	0.79 (0.41)	*F* (2,781) = 3.37	0.035
Presence of romantic partner	0.11 (0.31)	0.09 (0.28)	0.18 (0.38)	*F* (2,781) = 6.89	0.001

The self-esteem of girls showed a very slight decrease from T1 to T2 (from 3.08 to 3.04) and then a slight increase from T2 to T3 (from 3.04 to 3.07). Differences in daughters’ self-esteem over time were marginally significant (*p* = 0.065). A similar, although more pronounced, trend was visible for changes in the perceived quality of the father–daughter attachment relationship (from 4.19 to 4.09 between T1 and T2; from 4.09 to 4.16 from T2 to T3). These differences over time were significant (*p* = 0.033). The quality of perceived mother–daughter attachment relationship remained stable from T1 to T2 (4.54 at both waves), but showed a small increase from T2 to T3 (from 4.54 to 4.64). Differences in the perceived quality of the mother–daughter attachment relationship over time were significant (*p* = 0.002). Over time, and on average, adolescent girls perceived a small decrease in the quality of their parents’ relationship (from 4.20 to 4.13 between T1 and T2; and from 4.13 to 4.09 from T2 to T3). These differences over time were significant (*p* = 0.006). For adolescent girls, there was an increase in the number of close friends from T1 to T2 (from 13.76 to 16.33). From T2 to T3 however, the average number of friends a girl had dropped below the number it was at T1 (13.49 at T3). These differences over time were marginally significant (*p* = 0.052). For girls, the likelihood of having a best friend decreased over time, subsequently (from T1; 0.91 to T2; 0.85 to T3; 0.83), whereas the likelihood of having a romantic partner increased over time, from 0.13 to 0.16 from T1 to T2 and from 0.16 to 0.26 from T2 to T3. Both differences over time were significant (respectively *p* = 0.008 and *p* = 0.000).

Turning to the results for boys in Table 2, it can be seen that, in line with the existing literature, boys had higher levels of self-esteem on all three time points compared with girls: boys scored 3.30 (T1), 3.28 (T2) and 3.33 (T3) compared to 3.08 (T1), 3.04 (T2) and 3.07 (T3) for girls. However, the slope of self-esteem seems similar to that of girls; it showed a small decrease from T1 to T2 (from 3.30 to 3.28) and then slightly increased from T2 to T3 (from 3.28 to 3.33). In contrast to girls though, boys ended up with an averaged self-esteem level at T3 which was higher than at T1. In line with the findings for daughters, differences in sons’ self-esteem over time were marginally significant (*p* = 0.071). In contrast to the trend seen for girls, the perceived quality of the father–son attachment relationship became of continued lower quality over time, respectively 4.33 (T1), 4.22 (T2) and 4.19 (T3). Differences in the perceived quality of the father–son attachment relationship over time were significant (*p* = 0.006). The perceived quality of the mother–son attachment relationship experienced a decrease from T1 (4.53) to T2 (4.42), but then made an almost full recovery from T2 to T3 (4.50). These differences over time were significant (*p* = 0.031). Similar to the trend seen for girls, over time, adolescent boys perceived a continuing decrease in the quality of their parents’ relationship (from 4.24 at T1, to 4.21 at T2 to 4.15 at T3). These differences over time were also significant (*p* = 0.011). Over time, the number of close friends reported by adolescent boys increased steadily over time, from an averaged 16.53 to 18.37 and finally to 19.73. These differences over time were, however, not significant (*p* = 0.105). In line with the trend seen for girls, the likelihood of having a best friend decreased slightly over time for boys as well (from 0.85 to 0.82 to 0.79). Boys in general were less likely to report having a best friend in comparison to girls. Finally, the likelihood of having a romantic partner decreased slightly from T1 (0.11) to T2 (0.09), but made an increase from T2 to T3 (0.18). Both differences over time were significant (respectively *p* = 0.035 and *p* = 0.001).

### Multivariate results

The results from the fixed effect models are shown in Table 3 (daughters) and Table 4 (sons). The first model in Table 3 informs us that the changes in daughters’ perceived quality of the father–child attachment relationship were significantly linked with changes in daughters’ self-esteem (*b* = 0.14, *p* = 0.002). When the quality of the perceived father–child attachment relationship decreased over time (average pattern observed between T1 and T2), the level of daughters’ self-esteem also decreased. When the quality of the perceived father–child attachment relationship improved over time (average pattern observed between T2 and T3), the level of daughters’ self-esteem also increased. In addition, the current study found that when the quality of the perceived mother–child attachment relationship improved over time, daughters’ self-esteem improved in tandem (*b* = 0.16, *p* = 0.001). The second model solely included information on changes in the perceived parental relationship quality, changes in the number of close friends and changes in the presence of a best friend and a romantic partner. This study found that changes in parents’ relationship quality were significantly linked with changes in daughters’ self-esteem (*b* = 0.24, *p* = 0.000); when the quality of the parental relationship decreased over time, daughters self-esteem decreased in tandem. Contrary to expectations, changes in the number of close friends and changes in the presence of having a best friend were not significantly linked with changes in daughters’ self-esteem. Changes in the presence of a romantic partner, however, were marginally significantly positively linked with changes in girls’ self-esteem (*b* = 0.10, *p=* 0.058); becoming romantically involved increased girls’ level of self-esteem. In the third model, all variables from model 1 and 2 were included. Likelihood-ratio tests revealed that these additions improved model fit (From model 1 to model 3 *χ*^2^ = 18.36; from model 2 to model 3 *χ*^2^*=* 67.28). With the inclusion of information on changes in the perceived quality of the parental relationship, changes in the number of close friends, changes in the presence of a best friend and changes in the presence of a romantic partner, the strength of the coefficients for changes in the quality of the perceived father–child (*b* = 0.15, *p* = 0.008) and mother–child attachment relationship (*b* = 0.14, *p* = 0.006) remained highly similar and significant. The strength of the coefficient for changes in the perceived quality of the parental relationship dropped from *b* = 0.24 (*p* = 0.000) to *b* = 0.14 (*p* = 0.009), but remained significant.

**Table 3:** Fixed effects analyses for daughters (*N* = 276), *B* coefficients

	M1	M2	M3
	*B* (SE)	*B* (SE)	*B* (SE)
Change in quality father–child attachment relationship	0.14 (0.05)**		0.15 (0.06)**
Change in quality mother–child attachment relationship	0.16 (0.05)**		0.14 (0.05)**
Change in parents’ relationship quality		0.24 (0.05)***	0.14 (0.05)**
Change in number of close friends		−0.00 (0.00)	−0.00 (0.00)
Change in presence best friend		−0.02 (0.06)	−0.02 (0.06)
Change in presence romantic partner		0.10 (0.05)^#^	0.09 (0.05)^#^
Constant	1.75 (0.19)***	2.09 (0.22) ***	1.21 (0.26)***
Likelihood-ratio-test (compared with M3)	18.36***	67.28***	
*R* ^2^	0.10	0.06	0.14
AIC	381.1056	340.9582	277.6804

*AIC* Akaike information criterion

^#^*p* < 0.10; **p< 0.01; ***p< 0.001

**Table 4:** Fixed effects analyses for sons (*N* = 266), *B* coefficients

	M1	M2	M3
	*B* (SE)	*B* (SE)	*B* (SE)
Change in quality father–child attachment relationship	0.07 (0.04)^#^		0.06 (0.05)
Change in quality mother–child attachment relationship	0.15 (0.05)**		0.13 (0.05)*
Change in parents’ relationship quality		0.19 (0.05)***	0.11 (0.05)*
Change in number of close friends		0.00 (0.00)	−0.00 (0.00)
Change in presence best friend		−0.04 (0.05)	−0.06 (0.05)
Change in presence romantic partner		−0.05 (0.07)	−0.03 (0.07)
Constant	2.32 (0.16)***	2.56 (0.21)***	2.05 (0.24)***
Likelihood-ratio-test (compared with M3)	9.87*	35.73***	
*R* ^2^	0.07	0.04	0.08
AIC	222.747	190.4028	158.6771

*AIC* Akaike information criterion

^#^*p* < 0.10; *p < 0.05; **p < 0.01; ***p < 0.001

In Table 4, attention is shifted to adolescent sons. The first model showed that changes in the perceived quality of the father–child attachment relationship were positively and significantly linked with changes in adolescent sons’ self-esteem, albeit only marginally (*b* = 0.07, *p* = 0.099): Decreases in the perceived quality of the father–child attachment relationship were associated with decreases in son’s self-esteem. Changes in the quality of the mother–child attachment relationship showed strong positive linkages with changes in son’s self-esteem (*b* = 0.15, *p* = 0.003). When the quality of the perceived mother–child attachment relationship decreased over time (average pattern observed between T1 and T2), the level of sons’ self-esteem also decreased. When the quality of the perceived mother–child attachment relationship improved over time (average pattern observed between T2 and T3), the level of sons’ self-esteem also increased. The second model solely incorporated information on parents’ relationship quality, the number of close friends, the presence of a best friend and the presence of a romantic partner. Similar to the finding for daughters, this study also saw for sons that changes in parents’ relationship quality were significantly linked with changes in sons’ self-esteem (*b* = 0.19, *p* = 0.000); when the quality of the parental relationship as perceived by sons decreased over time, sons’ self-esteem decreased in tandem. Contrary to the expectations however, changes in none of the peer characteristics were significantly linked with changes in sons’ self-esteem. In the third model, all variables from model 1 and 2 were included. Likelihood-ratio tests revealed that these additions improved model fit (From model 1 to model 3 *χ*^2^*=* 9.87; from model 2 to model 3 *χ*^2^*=* 35.73). With the inclusion of information on changes in the perceived quality of the parental relationship, changes in the number of close friends, changes in the presence of a best friend and changes in the presence of a romantic partner, changes in the perceived quality of the father–child attachment relationship were no longer significantly linked with changes in adolescent sons’ self-esteem (*b* = 0.06, *p* = 0.184). Linkages between changes in the perceived mother–child attachment relationship and changes in son’s self-esteem weakened by the inclusion of these variables, but remained strong and significant (*b* = 0.13, *p* = 0.013). The strength of the coefficient for changes in the perceived quality of the parental relationship dropped from *b* = 0.19 (*p* = 0.000) to *b* = 0.11 (*p* = 0.028), but remained significant.

### Sensitivity analyses and alternative analyses

In additional analyses, this study examined whether the differences found between the models for daughters and sons differed significantly from each other. For all the specific pathways, interaction effects with gender of the child were conducted. Results showed that the impact of changes in the perceived quality of the father–child attachment relationship on changes in adolescent’s self-esteem differed significantly between daughters and sons (coefficient for the interaction effect: *b* = 0.10; *p* = 0.04; coefficients for main effect of attachment to father: *b* = 0.10; *p* = 0.185). Coefficients for all other independent variables did not differ significantly between daughters and sons. Results from the full model are available upon request.

Furthermore, in a subsample of adolescents who had a romantic partner on any of the three waves this study examined whether changes in the quality of the romantic relationship were related to changes in adolescents’ self-esteem. These analyses were not included in this study’s main findings, as the majority of the sample was not involved in a romantic relationship on any of the three time points in this study. Choosing to focus on this variable would have led to a significant reduction of the sample size. Furthermore, adolescents could be in different relationships at the three different time points, which may make it relatively more difficult to interpret the hypothesized linkages between changes in the quality of the romantic relationship and changes in adolescent’s self-esteem. Nevertheless, in additional analyses, linkages between changes in the quality of the romantic relationship and changes in adolescent’s self-esteem were examined. In contrast to the expectations, this study did not find any significant relations between changes in the quality of the romantic relationship and changes in self-esteem, not for daughters (*b* = −0.10; *p* = 0.12), nor sons (*b* = −0.14; *p* = 0.35). Results from the full model are available upon request.

This study also ran additional analyses which tested whether changes in the wider family and social contexts of the adolescents, in specific changes in the parental relationship, changes in the number of close friends, changes in the presence of a best friend and changes in the presence of a romantic partner had an influence on linkages between changes in the perceived quality of the father–child and mother–child attachment relationship and changes in adolescents’ self-esteem. Because none of these interactions were significant (results available upon request) and because this article was deemed to be already quite complex, it was decided to leave the focus on these interaction effects out of the current article.

## Discussion

Studies have frequently underscored the importance of parent–child attachment relationships for adolescents’ self-esteem. However, more information regarding the unique contributions of father-child and mother–child attachment relationships on adolescents’ self-esteem is needed, not least for intervention purposes. Using self-reports of 542 adolescents from all three waves of the Dutch cohort study Social Development of Adolescents were used, this study showed that, for both sons and daughters, changes in the perceived quality of the mother–adolescent attachment relationship and changes in the perceived quality of the relationship between adolescents’ parents were positively linked with changes in self-esteem. Interestingly, changes in the perceived quality of the attachment relationship with father were only significantly linked to changes in daughters’ self-esteem, not to that of sons. In contrast with expectations, changes in peer relationships were not associated with changes in adolescents’ self-esteem. The finding that positive changes in the perceived quality of the attachment with mothers were related to a positive change in the self-esteem of both sons and daughters, indicates that mothers remain a primary attachment figure during adolescence and young adulthood (Rosenthal and Kobak 2010). The fact that this study also found linkages between perceived changes in the parenting relationship and changes in sons’ and daughters’ self-esteem and significant associations between changes in the perceived quality of the attachment relationship with father and daughters’ self-esteem, underscores the importance of including the entire family system in future interventions.

This study’s results showed that increases in the perceived quality of the mother–child attachment relationship were linked with increases in levels of self-esteem for both adolescent sons and daughters. These findings suggest that even though adolescents may be increasing their time spent with friends and romantic partners, mothers remain of strong importance during adolescence and young adulthood (Rosenthal and Kobak 2010). Changes in the perceived quality of the father–adolescent attachment relationship were only associated with daughters’ self-esteem. This latter finding is in line with the study of Liu (2008), which was performed in a non-Western society (i.e. Taiwan) with adolescents of comparable age as in the current study. For sons, the association between perceived changes in the quality of the attachment relationship with their father and changes in self-esteem was reduced to insignificance when changes in the perceived quality of the parental relationship were included in the model. This suggests that fathers’ potential for influencing their son’s self-esteem lies mainly within their role as part of the parental dyad, more specifically in the way that sons perceive the quality of the relationship of their parents.

Scholars have suggested that it is important to distinguish between father–child attachment relationships and mother–child attachment relationships because of the differential contributions they each make to adolescent psychological functioning and adjustment (e.g., Rice et al. 1997; Buist et al. 2002; Plunkett et al. 2007). This study underscores the importance of distinguishing between mother–son, father–son and mother–daughter and father–daughters dyads in order to provide a comprehensive understanding of whether and to what extent there are different ways in which mothers and fathers contribute to the perceived attachment relationship with their children and subsequently contribute to their adolescent children’s self-esteem.

In the words of Lieberman et al. (1999), *there appears to be some uniqueness to the father–daughter [attachment] relationship as girls approach adolescence* (p. 209). The current study’s findings underscore this observation. Theories of gender (e.g., West and Zimmerman 1987) suggest that societal beliefs systems encourage females to be supportive and interdependent and males are urged to be competitive and solve problems. As such, boys have been encouraged from a young age to be independent, confident and competitive. Daughters, in contrast, may in particular start to value their fathers’ independence-encouraging behavior in adolescence, which shapes the way they respond to changes in the perceived quality of the father–child attachment relationship. The current study wasn’t able to put this hypothesis to the empirical test. Future research should shed light on the possible underlying mechanisms which could explain why changes in perceived quality of the father–adolescent attachment relationship were related only to daughters’ and not son’s changes in self-esteem.

Beyond the influence of dyadic attachment relationships, this study found, for both daughters and well as sons, strong linkages between decreases in the perceived quality of the parental relationship and decreases in adolescents’ self-esteem. The strong and consistent contribution of parental relationship quality (Grych et al. 2000) and parental conflict (e.g., Turner and Kopiec 2006) underscores the need to use a family system perspective (Cox and Paley 1997) when analyzing the impact of parents on children’s developmental outcomes. Given that self-esteem has been linked with delinquency, substance abuse, depression, anger, and aggression (e.g., Emler 2001), it seems worthwhile to intervene when adolescents’ shown signs of low self-esteem. The current study suggest that clinicians should incorporate the parental relationship when looking for areas in which intervention could take place.

The current study did not find evidence for direct linkages between changes in peer relationships and changes in adolescents’ self-esteem. Although these findings are in line with findings from earlier studies (e.g., Paterson et al. 1994; Noom et al. 1999; Wilkinson and Walford 2001), they do contrast several studies that have shown that peer influences are significant related to adolescents’ self-esteem but only when parental relationships are left out of the equation (e.g., Bagwell et al. 1998; Bishop and Inderbitzen 1995; Keefe and Berndt 1996; Kingery et al. 2011; Luciano and Orth 2017; Murray et al. 2000). The fact that this study did not find any significant findings for changes in peer relationships on changes in adolescents’ self-esteem, might be related to its measurement of peer relationships. First and foremost, in the current study it wasn’t possible to measure perceived peer *attachment*. This study was only able to ask about changes in the presence of a best friends, changes in the presence of a romantic partner and changes in the number of close friends. Although the focus on specific peers and dyadic relationships is compatible with attachment theory, the current study only measured quantitative differences in peer relationships rather than a qualitative evaluation of the attachment relationship. Even though additional analyses were ran testing linkages between changes in the quality of the romantic relationship and changes in adolescents’ self-esteem, these analyses were not without limitations. Future studies should ideally incorporate changes in the perceived quality of the attachment relationship with one’s best friend or romantic partner.

The current study focused on a global construct of self-esteem. There is now accumulative evidence that this construct consists of two dimensions: ability and worth (Brown 1998; Tafarodi and Milne 2002). Ability relates to the amount of “self-competence” that the individual feels, while worth relates to the extent of “self-liking” that the individual experiences. This study found evidence for the claim that attachment relationships with both mothers and fathers remain to be of importance in adolescence for children’s global construct of self-esteem. Given the current finding that linkages between changes in the perceived quality of attachment relationships with fathers and mothers and changes in self-esteem differ by adolescents’ gender, it would be interesting to investigate to what extent attachment relationships with mothers and fathers play different roles in the development of the two different aspects of self-esteem for sons and daughters.

In contemplating the findings, specifics of Dutch society should be noted. The Netherlands have the highest share of part-time working women of all European countries (Portegijs and Keuzenkamp 2008). Moreover, in the Netherlands a rather strong male breadwinner ideology coincides with a strong motherhood ideology; most Dutch men and women agree with the statement that fathers and not mothers should work full-time and that mothers are better at taking care of their children than are fathers (Portegijs and Merens 2010). Dutch men spend the least time with their children of all OECD countries (with the exception of Austria) (Fatherhood Institute 2010). Dutch mothers spend, on average, twice at much time with their children as Dutch fathers do (Portegijs and Merens 2010). In this light, it might not come as a surprise to the reader that changes in the perceived quality of the attachment relationship with mother are strongly related to sons’ and daughters’ changes in self-esteem. Nevertheless, it is encouraging to see that even in a country in which fathers are relatively weakly involved in childcare, a small increase in the perceived quality of the attachment relationship with father is linked with an increase in the self-esteem of their adolescent daughters.

This study was not without limitations. First, the findings of this study relied on adolescent self-report, which runs the risk of social desirability. Despite the fact that this is a common methodology for assessing adolescent attachment relationships, readers should recognize that the findings solely reflect adolescents’ states of mind regarding parental attachment relationships. Nonetheless, the findings from this study are consistent with both adolescent attachment theory and previous studies. Furthermore, adolescent’s developing cognitive capacities enable them to evaluate past attachment experiences as their own states of mind regarding attachment in a coherent way (Allen 2016). A further limitation of the current study is the fact that we, despite our longitudinal fixed effects research design, cannot rule our reversed causality. Changes in self-esteem may elicit changes in the perception of the quality of the attachment relationship with one’s parents. Although longitudinal data can assess congruent changes in the perceived quality of attachment relationships and attachment outcomes, it cannot firmly establish causality. However, the current study does indicate that changes in the perceived quality of attachment relationships with fathers and mothers are significantly related to changes in adolescents’ self-esteem. Because these variables may be reciprocally related, future research should ideally utilize experimental designs for revealing causality. A third limitation of this study pertains to the characteristics of this study’s sample. This study examined an ethnically homogeneous sample of middle-class Dutch families. These results may therefore not be generalized to an ethnically more diverse sample.

## Conclusion

Although there is consensus in the literature that parent–child attachment relationships are strongly related to adolescents’ self-esteem, answers to the question whether and to what extent fathers and mothers uniquely contribute to adolescents’ self-esteem have been equivocal. The current study separated the potential influences of father–child and mother–child attachment relationships on sons’ and daughters’ self-esteem, controlled for changes in the quality of the parental relationship and peer relationships and used longitudinal fixed effects models to isolate linkages between changes in adolescents’ perceived quality of the parent–child attachment relationships and changes in adolescents’ self-esteem. The study showed that changes in the perceived quality of the mother–child relationship and changes in the perceived quality of the parental relationship matter for both sons’ and daughters’ self-esteem, whereas changes in the perceived quality of the father–child attachment relationship is only linked to adolescent daughters’ self-esteem. As such, these findings shed more light on the potential for future interventions to boost adolescents’ sense of self-worth. Up to the 1970s attachment studies mostly focused on the influence of mothers on child development. Since then more studies have shown the importance of the role of the father (Grossman et al. 2002; Leidy et al. 2013; Panter-Brick et al. 2014; Paquette 2004; Vékony et al. 2004). This study underscores the importance of including the entire family system in future interventions when one aims to improve adolescents’ self-esteem. Furthermore, this study highlights that fathers have an impact on their daughters’ self-esteem beyond that of mothers. Given that in adolescence in particular girls report lowered self-esteem, it is important that policy and program makers do not overlook the role of fathers.
